# Authors’ reply - anaphylactic shock with methylprednisolone, Kounis syndrome and Hypersitivity to corticosteroids: a clinical paradox

**DOI:** 10.1186/s13052-018-0600-z

**Published:** 2019-01-07

**Authors:** F. Porcaro, M. G. Paglietti, A. Diamanti, F. Petreschi, A. Schiavino, V. Negro, V. Pecora, A. Fiocchi, R. Cutrera

**Affiliations:** 1 Respiratory Unit, Academic Department of Pediatrics, Bambino Gesù Children’s Hospital, IRCCS, Piazza di Sant’Onofrio 4, 00165 Rome, Italy; 2 Artificial Nutrition Unit, Bambino Gesù Children’s Hospital, IRCCS, Rome, Italy; 3 Division of Allergy, Academic Department of Pediatrics, Bambino Gesù Children’s Hospital, IRCCS, Rome, Italy

**Keywords:** Anaphylactic shock, Corticosteroids, Kounis syndrome

## Abstract

In our letter, we comment the paper of Kounis et al., that highlights a poor-known clinical entity determined by systemic use of corticosteroids, the so-called “Kounis syndrome type I”. We appreciated and shared the intent of Authors to treat the important issue of high risk of adverse drug reaction in patients with atopic diathesis and we confirm the need to administer corticosteroids with caution in patients suffering from allergic disease.

## Letter to the Editor

Dear Editor,

in their comment to our case report on a one-year-old male presenting anaphylactic shock episodes triggered by intravenous administration of methylprednisolone sodium succinatum, Kounis et al., highlighted a poor-known clinical entity determined by systemic use of corticosteroids (the so-called “Kounis syndrome type I”) [1]. Authors have furtherly raised the important issue of high risk of adverse drug reaction in patients with atopic diathesis.

We thank the Authors for having provided a comprehensive overview on pharmacodynamic profile of corticosteroids and the main mechanisms underpinning the most frequent adverse reactions to these common-used drugs.

As already exhaustively reported, two type of hypersensitivity reactions to systemic steroid administration should be mainly considered: immediate (IgE-mediated) and delayed (T cell-mediated) reactions [2].

Because of the risk of life-threatening reactions, to investigate mechanisms underlying immediate steroid allergic reactions is of great interest into scientific community.

The native drug-molecule or a drug metabolite (hapten) may act as an allergen. Moreover, esters used to create soluble injectable preparations (sodium succinate and sodium phosphate) can, sometimes, justify the allergic reactions rather than the glucocorticoid itself. Finally, glucocorticoid preparations may contain preservatives and excipients (e.g. lactose, carboxymethylcellulose, polyethylene glycol, hexylene glycol) to which sensitized patients can react [2].

In our case report, the anaphylactic shock occurred in a cow’s milk allergic patient, after the administration of systemic steroid formulation containing lactose and cow’s milk proteins traces. Although the first allergic reaction was attributed to the antibiotic treatment, the next episode clarified the cause-effect relationship allowing the diagnosis.

The delayed diagnosis of immediate allergic reaction to steroid is another important issue. In fact, patients typically receive glucocorticoids for several clinical conditions (asthma, anaphylaxis or shock) which present signs and symptoms similar to those of allergic reactions. Therefore, it can be difficult to distinguish a progressively worsening of medical condition from a superimposed allergic reaction to a glucocorticoid.

In conclusion, we agree with the Authors on the need to administer corticosteroids with caution in patients suffering from atopic disease.
